# Smartphone-Enabled Health Coaching Intervention (iMOVE) to Promote Long-Term Maintenance of Physical Activity in Breast Cancer Survivors: Protocol for a Feasibility Pilot Randomized Controlled Trial

**DOI:** 10.2196/resprot.6615

**Published:** 2017-08-24

**Authors:** Paul Ritvo, Maya Obadia, Daniel Santa Mina, Shabbir Alibhai, Catherine Sabiston, Paul Oh, Kristin Campbell, David McCready, Leslie Auger, Jennifer Michelle Jones

**Affiliations:** ^1^ School of Kinesiology and Health Science York University Toronto, ON Canada; ^2^ Department of Psychology University of Toronto Toronto, ON Canada; ^3^ Cancer Rehabilitation and Survivorship Program Princess Margaret Cancer Centre University Health Network Toronto, ON Canada; ^4^ Kinesiology and Physical Education University of Toronto Toronto, ON Canada; ^5^ General Internal Medicine University Health Network Toronto, ON Canada; ^6^ Department of Medicine University of Toronto Toronto, ON Canada; ^7^ Cardiovascular Prevention and Rehabilitation Program Toronto Rehabilitation Institute University Health Network Toronto, ON Canada; ^8^ Pharmacology and Toxicology University of Toronto Toronto, ON Canada; ^9^ Department of Physical Therapy University of British Columbia Vancouver, BC Canada; ^10^ Surgical Oncology University Health Network Toronto, ON Canada; ^11^ Division of Surgery University of Toronto Toronto, ON Canada; ^12^ Kinesiology Program University of Guelph—Humber Toronto, ON Canada; ^13^ Department of Psychiatry University of Toronto Toronto, ON Canada

**Keywords:** breast neoplasm, exercise, health coaching, RCT, telehealth

## Abstract

**Background:**

Although physical activity has been shown to contribute to long-term disease control and health in breast cancer survivors, a majority of breast cancer survivors do not meet physical activity guidelines. Past research has focused on promoting physical activity components for short-term breast cancer survivor benefits, but insufficient attention has been devoted to long-term outcomes and sustained exercise adherence. We are assessing a health coach intervention (iMOVE) that uses mobile technology to increase and sustain physical activity maintenance in initially inactive breast cancer survivors.

**Objective:**

This pilot randomized controlled trial (RCT) is an initial step in evaluating the iMOVE intervention and will inform development of a full-scale pragmatic RCT.

**Methods:**

We will enroll 107 physically inactive breast cancer survivors and randomly assign them to intervention or control groups at the University Health Network, a tertiary cancer care center in Toronto, Canada. Participants will be women (age 18 to 74 years) stratified by age (55 years and older/younger than 55 years) and adjuvant hormone therapy (AHT) exposure (AHT vs no AHT) following breast cancer treatment with no metastases or recurrence who report less than 60 minutes of preplanned physical activity per week. Both intervention and control groups receive the 12-week physical activity program with weekly group sessions and an individualized, progressive, home-based exercise program. The intervention group will additionally receive (1) 10 telephone-based health coaching sessions, (2) smartphone with data plan, if needed, (3) supportive health tracking software (Connected Wellness, NexJ Health Inc), and (4) a wearable step-counting device linked to a smartphone program.

**Results:**

We will be assessing recruitment rates; acceptability reflected in selective, semistructured interviews; and enrollment, retention, and adherence quantitative intervention markers as pilot outcome measures. The primary clinical outcome will be directly measured peak oxygen consumption. Secondary clinical outcomes include health-related quality of life and anthropometric measures. All outcome measures are administered at baseline, after exercise program (month 3), and 6 months after program (month 9).

**Conclusions:**

This pilot RCT will inform full-scale RCT planning. We will assess pilot procedures and interventions and collect preliminary effect estimates.

**Trial Registration:**

ClinicalTrials.gov NCT02620735; https://clinicaltrials.gov/ct2/show/NCT02620735 (Archived by WebCite at https://clinicaltrials.gov/ct2/show/NCT02620735)

## Introduction

### Background

Breast cancer, the most frequently diagnosed female cancer, accounts for approximately 25% of new Canadian cancer diagnoses [1]. With improvements in early detection and treatment, breast cancer mortality has decreased significantly in the last 30 years despite increasing incidence [2,3]. Most breast cancer cases diagnosed at localized stages are associated with a mean 5-year survival rate of 96% [2,4].

Despite these improvements, breast cancer treatments can result in the long-term effects of chronic pain, fatigue, neuropathy, functional limitations, sleep disturbance, sexual dysfunction, infertility, cognitive impairment, cardiorespiratory dysfunctions, and generally reduced well-being [5-14]. Breast cancer survivors also confront elevated risks for local or distal recurrence, metastases, second primary cancers, type 2 diabetes, and cardiovascular disorders [3,15-23].

Physical activity can improve cancer outcomes and quality of life while reducing adverse effects and risks. Moderate-to-vigorous physical activity (MVPA) during or after breast cancer treatment is specifically associated with reductions in cancer-specific and all-cause mortality [23,24]. As reported in a recent systematic review of 17 breast cancer–specific observational studies, breast cancer–specific mortality reductions of 13% to 51% were observed when the highest-to-lowest physical activity categories were compared [25-32].

The American College of Sports Medicine (ACSM), the American Cancer Society, and Worldwide Cancer Research (among other national and international agencies) recommend 150 minutes per week of moderate intensity physical activity for cancer survivors [33-37]. While most breast cancer survivors believe in exercise benefits [37], physical activity levels generally reduce after a breast cancer diagnosis with the large majority of breast cancer survivors (more than 80%) not meeting recommended physical activity levels [29,37-50].

Nonetheless, the impact of a cancer diagnosis often stimulates patients to reconsider lifestyle modification [43,46,50-52], providing clinicians the opportunity to introduce physical activity promotion [53]. Substantial evidence supports the efficacy of several intervention approaches in short-term physical activity change [54-57], with findings from systematic reviews and meta-analyses of exercise studies involving cancer survivors indicating that MVPA (1) is safe and well-tolerated [35,58], (2) can significantly improve quality of life [58-61] and, (3) can improve aerobic and musculoskeletal fitness, body composition, social functioning, and mental health and reduce fatigue [36,55,56,58,61-69].

MVPA benefits following breast cancer diagnosis are only maintained for as long as exercise behaviors continue [70-72]. Therefore, the longitudinal assessment of MVPA maintenance following interventions is critical [57,72,73]. In noncancer populations, physical activity intervention effects are infrequently maintained [74-84].

Despite varying reports of barriers, the long-term maintenance of and adherence to MVPA protocols in cancer survivors has not been adequately studied [74-84]. For example, in a recent systematic review of physical and/or dietary interventions in breast cancer populations [85], only 10 of 63 trials assessed the postintervention maintenance of behavioral outcomes [85]. Of these, 4 of 10 achieved successful maintenance (defined as longer than 3 months) [85]. In one recent study of 488 long-term (more than 5 years) cancer survivors with mixed tumor types, participants were randomized to a wait-list control or to a combined diet and physical activity intervention consisting of mailed print material and 15 telephone counseling sessions over 12 months. In the intervention group, weekly physical activity levels increased significantly from baseline (37.5 minutes) to 1-year assessment (postintervention, 101.0 minutes), and these elevated levels were maintained at the 2-year follow-up assessment [86]. While these findings are encouraging, reliance on self-reported physical activity measures and low recruitment rates warrant additional studies with improved designs. Altogether, longitudinal assessments of physical activity maintenance using objective measures in breast cancer survivors are rare, and more are needed to inform physical activity interventions aimed at achieving stabilized, long-term health outcomes [85,87].

Not surprisingly, the health behavior change methods guiding counseling in long-term MVPA maintenance have been inadequately tested in breast cancer survivors. Patient-centered interventions affecting multiple factors (eg, intrinsic motivations, perceived costs and benefits, barriers, ability to change) [88,89] have been derived from evidence-based models (eg, transtheoretical model, social cognitive theory, cognitive behavior theory, and theory of planned behavior) and, in the past, their related efficacies in changing multiple lifestyle behaviors (eg, smoking, diet, chronic sedentariness) have been demonstrated [87,90-93]. While evidential support does not favor one behavior change model, successful physical activity promotion programs have included self-directed physical activity guided by a counselor, follow-up behavioral prompts [56,94-99], and more than 4 sessions of related counseling.

Implementation of theory-based behavior change models for breast cancer survivors aimed at longitudinally maintained MVPA must account for treatment-related sequelae, including adaptations that distract from or discourage health behaviors (eg, avoiding physical pain and discomfort). Accordingly, healthy physical activity promotion requires a cognitive component that emphasizes protective MVPA effects (eg, prevention of breast cancer recurrence) and a cognitive-behavioral component that assists incremental physical activity increases. Exercise prescriptions identify protective goals while carefully incremented training programs assist breast cancer survivors with immediate experiences of improved fitness, well-being, and achievement.

Counseling strategies for improving MVPA can benefit from Internet linkage, smartphone use, and wearable technologies. As of 2012, high proportions of Canadian households access broadband Internet, with mobile services adopted by nearly 80% [100]. Concomittantly, smartphone use has increased from 33% to 56% in all adult Canadians [101]. In the United States, by 2011 78% of adults used the Internet [102] and at least 64% use smartphones [103]. With increasing use, mobile technology has a rapidly increasing health care role via clinical decision making and data collection supporting chronic disease self-management [102].

In support of health behavior change, Internet linkage can provide timely reminders, assessments, behavior-tracking, and “just in time” reinforcement [101]. Supportive communications between patients and providers can occur during the critical periods of dynamic change rather than hours, days, weeks, or months later. Wearable fitness technologies have become more user-friendly with integrated feedback [104] accessed through mobile devices and Internet-linked computers [105] with reliable monitoring of physical activity at lower costs than research accelerometers [106]. Although few in number, Fitbit studies have reported 95% to 99% validity when Fitbit step counts (measured through smartphone apps) are compared with directly measured steps in healthy participants and stroke [106] and traumatic brain injury patients [107].

Despite the accumulating evidence of improved health outcomes with mobile technologies in diabetes, asthma, cardiovascular disease [102,103,108], and physical activity promotion [108], these technologies have been understudied in cancer populations [109]. To advance adoption of long-term physical activity in breast cancer survivors, our innovative health coaching intervention (iMOVE) includes applications of smartphone, computer, and wearable technologies. The pilot study will evaluate iMOVE and inform the design of a larger pragmatic randomized controlled trial (RCT).

### Aims of the Pilot Study

Aim 1: To evaluate recruitment, retention, and adherence with a goal of recruiting more than 40% of eligible, contacted patients, retaining more than 75% of participants until the 6-month assessment, and seeing more than 70% of intervention components completed.

Aim 2: To evaluate acceptability feedback for intervention modification in the anticipated full-scale RCT.

Aim 3: To determine pilot estimates of intervention efficacy on fitness (primary outcome) and patient-reported, anthropometric, physical, and psychosocial outcomes (secondary outcomes).

## Methods

### Recruitment

This pilot RCT will enroll physically inactive breast cancer survivors stratified by age (55 years and older/younger than 55 years) and adjuvant hormone therapy (AHT) exposure (AHT or no AHT). Recruitment will be undertaken through the Princess Margaret Cancer Centre (PMCC), and interventions will occur at the Electronic Living Laboratory for Interdisciplinary Cancer Survivorship Research (ELLICSR), the Cancer Survivorship and Wellness Centre located at the Toronto General Hospital. Both institutions are members of the University Health Network in Toronto, Ontario, and research ethics board approval was obtained from the University Health Network (13-6157-DE). The trial is registered at ClinicalTrials.gov [NCT02620735].

### Participants

Adult (aged 18 to 75 years) female breast cancer survivors deemed disease-free after primary cancer treatment are eligible. See Textbox 1 for selection criteria.

### Recruitment and Randomization

After identification from weekly generated clinic lists and chart reviews, patients will be approached by a member of their clinical team, and interested patients will meet with a research assistant for additional study explanation and eligibility screening. Participants will also be recruited by advertisement flyers located in hospital common areas. Eligibility will be ascertained in person when possible, with written consent obtained in person prior to randomization. After participants complete baseline questionnaires and initial physiological assessments, stratification-related data (age, AHT status) will be emailed to a biostatistician in the Department of Biostatistics at PMCC who will perform randomization and then send a study identification with intervention or control group allocation.

**Inclusion and exclusion criteria for study.**

Inclusion criteria:

- Less than 2 years of completion of adjuvant therapy with the exception of hormone therapy for stage 0 to IIIA
- Self-report of fewer than 60 minutes of weekly preplanned physical activity
- Physician clearance for moderate-to-vigorous physical activity
- English proficiency
- Ability to attend exercise training sessions and study assessments at prescribed intervals for 9 months

Exclusion criteria:

- Plans to join a weight loss or exercise program within 9 months
- Current pregnancy or planned pregnancy within 9 months
- Planned surgery during study duration
- Unwillingness to be randomized

**Figure 1:**
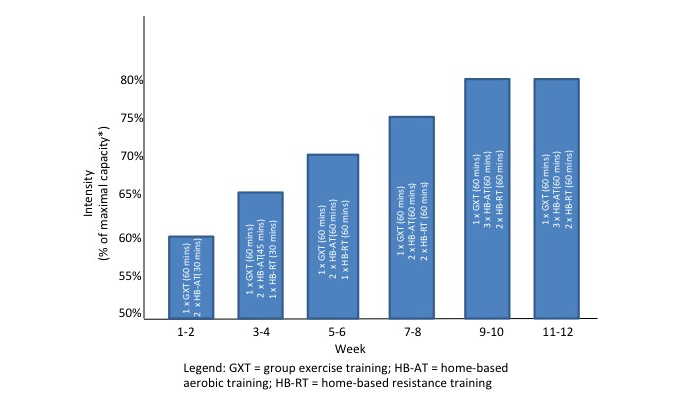
Exercise volume progression.

### Description of Treatment Arms

#### Exercise Training Program (Intervention and Control Arms)

Participants in the intervention and control groups will receive individualized exercise programming progressing toward the ACSM guidelines of 150 minutes per week of moderate intensity aerobic exercise, 2 to 3 days of resistance training, and routine flexibility training.

In the first 12 weeks, intervention and control participants have 1 supervised, facility-based group exercise class plus instruction for 2 additional unsupervised, home-based exercise sessions. Facility-based sessions will be offered at a variety of times and days weekly to accommodate schedules and increase accessibility. From weeks 13 to 26, participants will complete 3 to 5 home-based exercise sessions per week and no facility-based sessions. Each exercise program is individualized based on the initial fitness assessment, physical limitations, and exercise preferences. The exercise prescriptions are developed and monitored by a certified exercise physiologist (CEP) and a registered kinesiologist (RKin) with volume progression over the intervention course (see Figure 1). Facility-based aerobic training consists of low-impact exercises or aerobic training machines. Home-based aerobic training includes participant-selected aerobic exercises including brisk walking and cycling. Resistance exercise (facility- and home-based) is completed using resistance bands and stability balls provided to participants. The training intensity is based on exercise performance during group sessions observed by the CEP and RKin and is self-monitored via the 20-point Borg Scale for Rating of Perceived Exertion, with a target zone of 14 to 15, varying in accord with the subjective exercise experience of each participant. Adaptations to the exercise prescription based on the participant’s experiences, preferences, and changes (improvements or decrements) in physical capacity will be made by the CEP and RKin to optimize intervention efficacy and safety. All participants receive exercise manuals with exercise descriptions (eg, instructive photographs, exercise safety guidelines, stretching instructions) and a weekly exercise log to review with the CEP and RKin. This program is based on the ACSM guidelines [36] and modeled after a successful theory-based program developed by research team members [110-112].

#### iMOVE Health Coach (Intervention Arm)

Intervention group participants are additionally provided with a technology-enabled health coach intervention (iMOVE) with 3 components: one-on-one telephone-based counseling, supportive health tracking smartphone software (Connected Wellness, NexJ Health Inc), and use of Fitbit and associated software (FitBit Flex, Fitbit Inc). The iMOVE intervention is intended to enhance sustained behavior change (physical activity) by integrating several active ingredients outlined in the cancer-survivor behavior change literature [113,114] and based on multiple theories, specifically motivational interviewing (MI) [115], cognitive behavioral therapy (CBT) [116], and relapse prevention therapy [117,118].

Theoretical constructs focus on promoting motivation and establishing exercise self-efficacy, exercise social support, and positive exercise-related feelings during the acute intervention (12 weeks) that are sustainable during the postexercise program period (6 months). The telephone-based health coaching component of iMOVE includes 10 30-minute telephone calls with a trained health coach, scheduled at weeks 1, 2, 3, 4, 5, 6, 8, and 12 (during the exercise program) and at weeks 20 and 28 (postexercise program booster sessions). Health coaches with a counseling background in MI and CBT are trained and supervised by a registered clinical psychologist and a Motivational Interviewing Network of Trainers–certified trainer. The training of health coaches involves instructions on breast cancer and related survivorship issues and continual exposure to the multitheoretical approach. Training proceeds weekly from the trial start to end based on case review and participant responses to the approaches implemented. Each health coach call to participants focuses on the assessment and enhancement of motivation, promotion of self-efficacy, and collaborative problem solving. Telephone-based counseling provides several advantages over face-to-face, notably the potential to reach multiregional populations, as telephone access is widely available [119] and requires no user or provider transport. The schedule provides support while building autonomy and independent motivation [120]. See Textbox 2 for a theoretical base summary.

In the scheduled telephone-based sessions, participants will interact with the Connected Wellness software (NexJ Health Inc) by smartphone or Internet-linked computer. This software, previously found effective with participants diagnosed with type 2 diabetes [121-123], has now been tailored for breast cancer. It tracks physical activity, nutrition, pain, and psychological well-being (eg, mood, energy) and supports goal setting (with selective daily or hourly reminders). All software entries are time-stamped, allowing for graph creation that combines multiple trackers, enabling participants and health coaches to see change indicators in relation to the physical activity levels undertaken. Every initiation of contact by participants with their health coach via text messaging is recorded. Confirmations of received text messages are provided by coaches to participants. While patients are encouraged to further discuss their texts during the next phone session, there is also provision for the health coaches to text message responses immediately, responding to questions and issues raised.

Use of the Fitbit Flex provides further assistance to participants in adhering to recommended physical activity routines and tracking physical activity, notably providing real-time feedback (light-emitting diode device lights indicate percent completion of preset, daily step goals). Additional connectivity in the Health Coach program allows the participant and health coach to jointly explore daily physical activity experiences. Fitbit Flex vibrates when preset goals (eg, 10,000 steps) are reached and records the steps taken, combining them with user data to calculate distance walked, calories burned, and the duration and intensity of activity. Fitbit Flex also measures sleep quality by tracking periods of restlessness (ie, how long it takes the wearer to fall asleep per detected body movement) and the estimated sleep duration. The user can monitor their own activity on the Health Coach platform and create summaries and periodic analyses.

As is common with behavioral interventions, a handbook specifies sessional objectives and provides clinical tools for health coaches to use each session. The health coach creates a session-by-session agenda based on patient goals, monitored activity, and motivations as collected with the software during intervals between sessions and at each session. MI and CBT are the core health behavior change theories employed. MI is a collaborative counseling method that elicits and strengthens motivation for change by addressing and resolving ambivalence [124]. MI has demonstrated effectiveness in increasing physical activity in cancer survivors and those with other chronic conditions [41,95,125-132], and positive MI-related effects have been longitudinally detected (eg, at 2 years postintervention) [126,133]. In instances when self-efficacy is impeded by distorted cognitions, CBT principles will be applied, particularly to influence affect-balance through cognitive modifications that prevent or ameliorate negative mood fluctuations [117]. Telephone-based interventions have been effective and acceptable to breast cancer patients [134-137] and useful in delivering MI and CBT interventions [138-140]. Intervention fidelity will be assessed by routine reviews of implementation variables.

While there are multiple theoretical models integrated within our intervention, these models are consolidated in the focus on addressing and resolving motivational ambivalence and identification and modification of the cognitive distortions that maintain motivational ambivalence and prevent adoption of appropriate health behaviors.

**Theoretical base of iMOVE intervention.**

Multifactor focus:

- Intrinsic motivations
- Perceived costs-benefits
- Identification of barriers
- Abilities to change
- Exercise self-efficacy
- Exercise social support
- Positive exercise-related feelings

Application of evidence-based theory:

- Transtheoretical model
- Social cognitive theory
- Motivational interviewing
- Cognitive behavioral therapy
- Relapse prevention theory
- Theory of planned behavior

Successful physical activity promotion features:

- Self-directed physical activity with more than 4 sessions of counseling guidance
- Follow-up behavioral prompts

Unique tailoring to breast cancer survivors:

- Program pacing per treatment sequelae (eg, physical pain and discomfort)
- Cognitive emphasis on protective physical activity effects (eg, prevention of breast cancer recurrence)
- Cognitive behavioral emphasis on paced, regulated physical activity increases
- Flexible exercise prescription for protective goals and incremental increases that optimize fitness and well-being

Technological assistance:

- Rapidly increasing role for mobile technology in health management
- Enabling patient-provider contacts during critical periods
- User-friendly wearable technologies (95%-99% validity on step counts)

### Outcome Measures

#### Pilot Outcome Measures

These measures reflect appropriateness and effectiveness of design features:

1. Recruitment rate: based on Consolidated Standards of Reporting Trials criteria [141] via a screening log that enables data collection on eligible consented (pre- and post-initial screen) and eligible but nonrecruited individuals with nonrecruitment reasons documented.
2. Retention rate over the trial duration: the percentage of participants who complete the interventions and each data point; with reasons for drop out documented.
3. Capture of outcomes: recording of the proportion of participants at each time assessment point with complete or missing data.
4. Treatment implementation and fidelity: implementation of telephone sessions for the intervention group will be assessed by use documentation of the health coaching techniques and tools and identified barriers. Data from the health coaching software is stored on secure server and used to measure and analyze self-report and health coach activity.
5. Acceptability: telephone interviews will be conducted with a randomly selected subsample (n=25) of intervention participants following intervention completion. The goals are to explore participant perspectives of intervention feasibility and acceptability and to gain an understanding of experiences among those successful and unsuccessful at physical activity maintenance over differing time periods (eg, during initial 3 months of intervention, 6 months of intervention, 9 months of follow-up). An interpretive descriptive qualitative methodology will be used [142], and a record of interview participation will be kept to distinguish participants from those who don’t participate. The semistructured interviews will be about 45 minutes in duration and preceded by verbal informed consent. Interviews will be audiorecorded and transcribed verbatim.

#### Clinical Outcomes

Measures for fitness (primary), self-report (secondary), and anthropometric and physical outcomes (exploratory outcomes) are repeated at baseline, T1 (immediately after exercise program, month 3), and T2 (6 months after exercise program, month 9).

##### Primary Clinical Outcome

Cardiorespiratory fitness will be assessed by a graded exercise test using the modified Bruce protocol [143]. Directly measured peak volume of oxygen (mL/kg/minute) and anaerobic threshold will be obtained using a metabolic cart (TrueOne 2400, Parvo Medics) with continuous gas exchange analysis during incremental treadmill walking to volitional peak capacity. Blood pressure and arterial oxygen saturations are measured at rest and during exercise. Absolute and relative test termination criteria are based on standardized guidelines[144].

##### Secondary Clinical Outcomes

We will gather preliminary data on a number of exploratory variables which have been identified as important to understanding the potential impact of the intervention on patient-relevant and clinically-relevant outcomes. They are being collected to examine whether they are feasible to collect in a larger trial and whether they are responsive (sensitive to change) to the intervention [145,146].

###### Patient-Reported Clinical Outcomes

1. Godin-Shepherd Leisure-Time Exercise Questionnaire: a brief validated 3-item questionnaire that asks respondents to report on typical weekly exercise habits [147]
2. Functional Assessment of Cancer Therapy—Breast: generic quality of life measured with 44 self-report items [148]
3. Spielberger's State-Trait Anxiety Inventory—State [149]: a widely used 20-item measure of state anxiety
4. Center for Epidemiological Studies—Depression Scale short form: a 10-item self-report measure of depression
5. Functional Assessment of Cancer Therapy—Fatigue subscale [150]: a 13-item measure of fatigue in cancer patients
6. Breast Cancer Prevention Trial Symptoms Scale: a 42-item scale to assess side effects associated with the treatment of breast cancer
7. Fear of Recurrence Questionnaire: assesses anxiety about breast cancer recurrence [151]
8. Physical Activity Group Environment Questionnaire [152]: assesses group cohesion during exercise
9. Brief Pain Inventory [153]: a widely used measure to rapidly assess the severity of pain and its impact on functioning
10. Multiple Intervention Satisfaction Survey: an investigator-generated instrument that facilitates intervention participants in rank-ordering discrete intervention components with respect to how helpful they are in achieving outcomes during study participation. Additional items facilitate participant suggestions for deleting intervention components deemed (by participants) as of negligible benefit.

###### Anthropometric Clinical Outcomes

1. Body composition is assessed via body mass index, waist circumference, and body fat percentage
2. Waist circumference is measured according to the World Health Organization protocol (midpoint between lowest rib and iliac crest)
3. Body fat percentage is measured using bioelectrical impedance analysis [144]
4. Grip strength is measured using a Jamar dynamometer according to the Canadian Society for Exercise Physiology 2004 protocol

## Results

### Primary-Secondary Outcome Assessment

Recruitment and retention rates will be assessed [154] with estimates for participants with complete data per outcome and time point divided by the total number of study participants. Interpretation of the interview output (acceptability) will be based on inductive and deductive analyses and use of the constant comparative method [155].

Variability of the main and interaction effects will be examined in the primary clinical outcome (cardiovascular fitness) and each secondary outcome using separate repeated measures analysis of covariance models with Bonferroni corrections applied to the models. Hedges’ *g* and associated confidence intervals [156] will be calculated as an estimate of the effect size both over time (within groups) and between groups [157]. Missing data will be evaluated on a case-by-case basis such that drop-outs will be excluded. Intention-to-treat (all consented subjects) analysis will employ a last observation carried forward approach to evaluate all data collected. Per protocol analysis will evaluate data on subjects who participated in 50% of group exercise sessions (comparing intervention with control subjects), while experimental subjects will have the additional criteria of participating in 50% of health coaching calls.

### Sample Size and Power

We previously conducted a simulation for a range of sample sizes and different SD values for precision of the treatment effect estimate. The precision of the estimate is represented by the inverse of the margin of error. Type I error was set at α=0.05 and power at 80%. From our simulation result, a sample size of 35 to 40 was at the elbow point of the curves, indicating the precision of estimates did not proportionally increase with a larger sample size. Therefore, our projected sample size is 80 participants (40 per arm) [158]. With an anticipated drop-out rate of up to 25% [73], we will recruit 107 participants and examine the variance in primary outcomes with precision (low standard error >0.1), while enabling further calculations of effect sizes for planning the phase 3 trial [154]. The large majority of women return to PMCC for follow-up appointments typically scheduled every 3 to 6 months. Based on data from the PMCC registry, eligibility criteria, and expected participation rates, we anticipate recruiting 8 to 10 participants per month. Study duration is estimated at 30 months.

### Interpretation of Results

Interpretation of the effect size and mean difference scores and calculation of the sample size for a larger RCT (fitness outcome) will be based on a minimally important clinical difference (MCD) of 3.5 mL/kg/minute (peak volume of oxygen) between the 2 experimental groups at the 6-month T2 assessment [159,160]. We regard the MCD as a small effect size [159]. With pilot results, we will better estimate small, medium, and large effect sizes for the planned (full-scale) RCT.

## Discussion

While current data suggest an important role for physical activity in disease control and the long-term health of cancer survivors, most breast cancer survivors are inactive. This discrepancy must be addressed with physical activity promotion that supports long-term exercise adherence. To date, research has focused on specific physical activity components linked to clinical benefits, but insufficient attention has been paid to factors influencing long-term physical activity maintenance. The current project employs a behavioral support intervention that assists breast cancer survivors in adopting physical activity and maintaining physical activity adherence. While multiple RCTs demonstrate effectiveness in physical activity participation during trial conduct, decreases in physical activity after trial conclusion are an important concern. It is not yet known the degree to which smartphone-enabled health coaching combined with wearable fitness technology can contribute to the lifestyle changes required for breast cancer survivors to maintain healthy physical activity over the longer term.

Our commitment to the devised intervention (combining phone-based health coaching, Fitbit step tracking, health tracking software, face-to-face exercise classes, and fitness testing) accepts the design limitation of being unable to identify which intervention components provide key contributions to significant effects; future studies may be needed to tease out what worked best in further streamlining the intervention. However, we have mitigated limitations by logging all phone counseling calls undertaken (registering time durations per call) and additionally itemizing and quantifying all use of the health tracking software, Connected Wellness (NexJ Health Inc). Furthermore, we track all Fitbit use, including use patterns per time period (day, week, and month). Additionally, use of the Multiple Intervention Satisfaction Survey facilitates each participant in subjectively ranking the intervention components on importance and suggesting deletions of components that have not been significantly helpful. These efforts will enable us to learn about the prioritization of intervention components from each subject’s perspective. Another limitation entails not knowing which allocations of staff time (to the intervention) represent a cost savings when compared to other physical activity promotion approaches. Therefore, we will carefully assess staff time, preparing for ascertaining this cost dimension in the future.

This pilot will document the implementation of the methods and intervention, preliminary outcomes, and acceptability of the interventions by qualitative interview. It will assess effect size in primary and multiple secondary outcomes with corresponding confidence intervals for more definitive sample size calculations. Although pilot results will provide a foundation for full-scale RCT planning, we anticipate challenges for which we currently have only partial or potential solutions. For example, we will only have suggestive data for assessing specific intervention components and for selecting the optimal subset for full RCT testing. Furthermore, as a pilot study, we are still refining the ultimate sample size of the planned full-scale RCT. Additionally, while control subjects receive an approximation of current standard care for exercise promotion (at ELLICSR), we cannot fully account for the attentional differences in intervention and control conditions. Nonetheless, this pilot is a distinct step forward in addressing a gap in the promotion of longer term exercise adherence for breast cancer survivors.

## Abbreviations

ACSM: American College of Sports Medicine
AHT: adjuvant hormone therapy
CBT: cognitive behavioral therapy
CEP: certified exercise physiologist
ELLICSR: Electronic Living Laboratory for Interdisciplinary Cancer Survivorship Research
MCD: minimally important clinical difference
MI: motivational interviewing
MVPA: moderate-to-vigorous physical activity
PMCC: Princess Margaret Cancer Centre
RCT: randomized controlled trial
RKin: registered kinesiologist
